# Fine Particulate Matter Constituents and Cardiopulmonary Mortality in a Heavily Polluted Chinese City

**DOI:** 10.1289/ehp.1103671

**Published:** 2012-01-03

**Authors:** Junji Cao, Hongmei Xu, Qun Xu, Bingheng Chen, Haidong Kan

**Affiliations:** 1 State Key Laboratory of Loess and Quaternary Geology (SKLLQG), Institute of Earth Environment, Chinese Academy of Sciences, Xi’an, China; 2 Department of Epidemiology and Health Statistics, Institute of Basic Medical Sciences, Chinese Academy of Medical Sciences, Peking Union Medical College, Beijing, China; 3 School of Public Health, Key Lab of Public Health Safety of the Ministry of Education, Fudan University, Shanghai, China; 4 G_RI^o^CE (Research Institute for the Changing Global Environment) and Fudan Tyndall Centre, Fudan University, Shanghai, China

**Keywords:** air pollution, chemical constituents, fine particulate matter, mortality, time-series studies

## Abstract

Background: Although ambient fine particulate matter (PM_2.5_; particulate matter ≤ 2.5 µm in aerodynamic diameter) has been linked to adverse human health effects, the chemical constituents that cause harm are unknown. To our knowledge, the health effects of PM_2.5_ constituents have not been reported for a developing country.

Objectives: We examined the short-term association between PM_2.5_ constituents and daily mortality in Xi’an, a heavily polluted Chinese city.

Methods: We obtained daily mortality data and daily concentrations of PM_2.5_, organic carbon (OC), elemental carbon (EC), and 10 water-soluble ions for 1 January 2004 through 31 December 2008. We also measured concentrations of fifteen elements 1 January 2006 through 31 December 2008. We analyzed the data using over-dispersed generalized linear Poisson models.

Results: During the study period, the mean daily average concentration of PM_2.5_ in Xi’an was 182.2 µg/m^3^. Major contributors to PM_2.5_ mass included OC, EC, sulfate, nitrate, and ammonium. After adjustment for PM_2.5_ mass, we found significant positive associations of total, cardiovascular, or respiratory mortality with OC, EC, ammonium, nitrate, chlorine ion, chlorine, and nickel for at least 1 lag day. Nitrate demonstrated stronger associations with total and cardiovascular mortality than PM_2.5_ mass. For a 1-day lag, interquartile range increases in PM_2.5_ mass and nitrate (114.9 and 15.4 µg/m^3^, respectively) were associated with 1.8% [95% confidence interval (CI): 0.8%, 2.8%] and 3.8% (95% CI: 1.7%, 5.9%) increases in total mortality.

Conclusions: Our findings suggest that PM_2.5_ constituents from the combustion of fossil fuel may have an appreciable influence on the health effects attributable to PM_2.5_ in Xi’an.

Numerous epidemiological studies during the past 20 years have confirmed that short- and long-term exposure to outdoor air pollution contributes to increased cardiopulmonary mortality and morbidity (Brunekreef and Holgate 2002; Pope and Dockery 2006). Among various pollutants in the ambient mixture, fine particulate matter (PM_2.5_; particles ≤ 2.5 µm in aerodynamic diameter) shows the most consistent association with adverse health outcomes and therefore is of great public health concern (Ito et al. 2011; Ostro et al. 2007; Peng et al. 2009; Thurston et al. 2005; Zhou et al. 2011). However, the chemical components of PM_2.5_ responsible for these effects are still unknown. As the U.S. National Academy of Science pointed out, it is important to understand the contributions of specific components of ambient particulate matter (PM) to cardiopulmonary and other health effects (National Research Council 1998).

China has one of the highest PM_2.5_ levels in the world (van Donkelaar et al. 2010). However, PM_2.5_ is still not a criteria pollutant in China, and few studies in the country have investigated the adverse health effects of PM_2.5_ because of a lack of monitoring data. Currently, the Chinese government is reviewing its Air Quality Standards (AQS) and proposing to set the annual and daily average PM_2.5_ standards as 35 µg/m^3^ and 75 µg/m^3^, respectively (Chinese Ministry of Environmental Protection 2010). To our knowledge, only three published studies have estimated the effects of PM_2.5_ on daily mortality in China (Kan et al. 2007; Ma et al. 2011; Venners et al. 2003). Kan et al. (2007) and Ma et al. (2011) found significant associations between PM_2.5_ and daily mortality in Shanghai and Shenyang, China, whereas Venners et al. (2003) observed negative but statistically insignificant associations between PM_2.5_ and daily mortality in Chongqing. Obviously, more studies are needed to investigate the health effects of PM_2.5_ and its chemical constituents in China.

In the present study, we examined short-term associations between PM_2.5_ constituents and cardiopulmonary mortality in Xi’an, a heavily polluted Chinese city.

## Methods

*Data.* Xi’an, with an area of 9,983 km^2^ and a resident population > 8.1 million in 2005, is the capital of Shanxi Province, China. Xi’an is the largest city in northwestern China, and it experiences some of the worst air pollution among China’s cities (Cao et al. 2005). Our study area was limited to the urban area of Xi’an, an area of 1,166 km^2^ with a resident population of > 2.7 million.

Mortality data. We obtained numbers of deaths among urban residents in Xi’an for each day for 1 January 2004 through 31 December 2008 from the Shanxi Provincial Center for Disease Control and Prevention (SPCDCP). In Xi’an, all deaths, regardless of whether they occur in a hospital or at home, must be reported to appropriate authorities before cremation of the remains. Hospital or community doctors must indicate the cause of death on a death certificate card that is sent to the SPCDCP. SPCDCP staff then classify the cause of death according to the *International Classification of Diseases, 10th Revision* [ICD-10; World Health Organization (WHO) 1992] as due to total nonaccidental causes (ICD-10 codes A00–R99), cardiovascular diseases (I00–I99), respiratory diseases (J00–J98), or injury (S00–T98). The Chinese government has mandated detailed quality assurance (QA) and quality control (QC) programs for the SPCDCP death registry.

Pollutant and meteorological data. For this study, we measured daily concentrations of PM_2.5_, organic carbon (OC), elemental carbon (EC), and 10 water-soluble ions [i.e., sodium ion (Na^+^), ammonium (NH_4_^+^), potassium ion (K^+^), magnesium ion (Mg^2+^) calcium ion (Ca^2+^), flouride (F^–^), choride (Cl^–^), nitrite (NO_2_^–^), sulfate (SO_4_^2–^) and nitrate (NO_3_^–^)] for 1 January 2004 through 31 December 2008 (1,827 days). We also measured concentrations of 15 elements [i.e., sulfur (S), chlorine (Cl), potassium (K), calcium (Ca), titanium (Ti), chromium (Cr), manganese (Mn), iron (Fe), nickel (Ni), zinc (Zn), arsenic (As), boron (Br), molybdenum (Mo), cadmium (Cd), and lead (Pb)] for 1 January 2006 through 31 December 2008 (1,096 days).

The PM_2.5_ monitoring site was located on the rooftop of the Chinese Academy of Sciences’ Institute of Earth Environment building in an urban-scale zone of representation (Chow et al. 2002). The site was surrounded by a residential area where there were no major industrial activities nor local fugitive dust sources [see Supplemental Material, Figure 1 (http://dx.doi.org/10.1289/ehp.1103671). PM_2.5_ samples were obtained 10 m above the ground. Our previous studies suggest that the measured PM_2.5_ concentrations at this monitoring station are representative of the general status of PM_2.5_ pollution in Xi’an (Cao et al. 2005, 2007, 2009).

**Figure 1:**
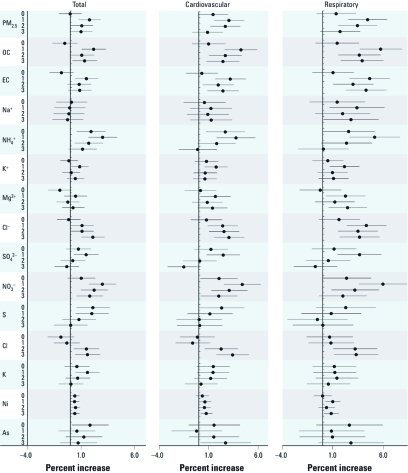
Estimated percent increases [mean (95% CI)] in total, cardiovascular, and respiratory mortality per IQR increase in pollutant concentrations on the current day (lag 0) or the previous 1–3 days (lags 1, 2, and 3), adjusted for temporal trend, day of the week, temperature, relative humidity, and SO_2_ and NO_2_ concentrations.

Daily PM_2.5_ samples were collected using two battery-powered mini-volume samplers (MiniVol™ TAS; Airmetrics, Eugene, OR, USA) operating at a flow rate of 5 L/min (Cao et al. 2003). We used a relatively low flow rate due to high PM loading in Xi’an. PM_2.5_ samples were collected on 47-mm Whatman quartz microfiber filters that were pre-heated at 900°C for 3 hr before sampling. The quartz-fiber filters were analyzed gravimetrically for mass concentrations. We analyzed a 0.5-cm^2^ punch from each sample for OC and EC using a Desert Research Institute (DRI) model 2001 thermal/optical carbon analyzer (Atmoslytic Inc., Calabasas, CA, USA) for eight carbon fractions following the IMPROVE (Interagency Monitoring of Protected Visual Environments) thermal/optical reflectance (TOR) protocol (Chow et al. 2004). Levels of the five water-soluble cations (Na^+^, NH_4_^+^, K^+^, Mg^2+^ and Ca^2+^) and five water-soluble anions (F^–^, Cl^–^, NO_2_^–^, SO_4_^2–^ and NO_3_^–^) were determined in aqueous extracts of the sample filters using an ion chromatograph (Dionex 600; Dionex, Thermo Fisher Scientific, Inc., Cambridge, England, UK). Cation concentrations were determined using a CS12A column (Dionex), and anions were separated by an AS11-HC column (Dionex). The elemental concentrations of these samples were then determined by energy dispersive X-ray fluorescence (ED-XRF) spectrometry using the PANalytical Epsilon 5 XRF analyzer (PANalytical B.V., Almelo, the Netherlands). Detailed descriptions of the sample pretreatment, specific methods, detection limits, and QA/QC have been discussed previously (Cao et al. 2003, 2005; Shen et al. 2009a, 2009b).

To adjust for the effect of gaseous pollutants and weather on mortality, we obtained daily concentrations of sulfur dioxide (SO_2_) and nitrogen dioxide (NO_2_) from the Xi’an Environmental Monitoring Center, and daily mean temperature and humidity from the Xi’an Meteorological Bureau. The SO_2_ and NO_2_ concentrations were averaged from the available monitoring results across seven stations in our study area. According to the rules of the Chinese government, we assumed the monitoring data from these stations generally reflected the background urban air pollution of Xi’an rather than pollution from local sources.

*Statistical methods.* Due to different time periods for measuring PM_2.5_ constituents, we constructed two data sets to analyze the data: The first involved daily measurement of PM_2.5_, OC, EC, and ions for 1 January 2004 through 31 December 2008 and the second included daily concentrations of PM_2.5_ and constituent elements for 1 January 2006 through 31 December 2008.

Daily counts of deaths and air pollution levels were linked by date and analyzed with time–series analyses (Bell et al. 2004). Because daily counts of deaths approximate a Poisson distribution and the relationship between mortality and explanatory variables is mostly nonlinear, we used overdispersed generalized linear Poisson models (quasi-likelihood) with natural spline (*ns*) smoothers to analyze mortality, PM_2.5_ constituents, and covariate data.

In the basic model, we incorporated smoothed spline functions of time, accommodating both nonlinear and nonmonotonic relations between mortality and time and thus providing a flexible model to control for long-term and seasonal trends (Hastie and Tibshirani 1990). Day of the week (*DOW*) was included as a dummy variable (a variable that takes on the values 1 and 0; also called an indicator variable) in the basic models. Partial autocorrelation function (*PACF*) was used to guide the selection of degrees of freedom (df) for the time trend until the absolute values of the sum of *PACF* of the residuals for lag days of up to 30 reached a minimal value (Peng et al. 2006; Touloumi et al. 2004, 2006). We used residual plots and *PACF* plots to examine residuals of the basic model for discernable patterns and autocorrelation.

After establishing the basic model, we introduced the PM_2.5_ constituents and covariates (including temperature, humidity, and SO_2_ and NO_2_ concentrations) in the model. Based on previous literature (Dominici et al. 2006), we used smoothed spline functions with 3 df (for the whole period of the study) to control for temperature and relative humidity. To examine the temporal relationship of PM_2.5_ constituents with mortality, we fitted the models with different lag structures from 0 lag days to 3 lag days because our previous work on PM_2.5_ and daily mortality in China showed little evidence of a significant association with a lag beyond 3 days (Kan et al. 2007; Ma et al. 2011). A lag of 0 days (lag 0) corresponds to the current-day PM_2.5_, and a lag of 1 day (lag 1) refers to the previous-day PM_2.5_. We used the smoothing spline, with 3 df for PM_2.5_, to graphically describe its relationships with mortality. We compared the linear and spline models by computing the difference between the deviances of the fitted two models (Dominici et al. 2002; Samoli et al. 2005). We estimated associations of PM_2.5_ constituents with mortality before and after adjustment for PM_2.5_ mass. Finally, to examine the robustness of our choice on the optimal values of df for time trend, we performed a sensitivity analysis to test the impact of df selection on the regression results.

All analyses were conducted in R version 2.10.1 (http://www.R-project.org) using the MGCV package. The results are presented as the percent change in daily mortality per interquartile range (IQR) increase of pollutant concentrations unless specified otherwise. Statistical significance was defined as *p* < 0.05.

## Results

We identified 47,838 deaths that occured between 1 January 2004 and 31 December 2008 in our study population. On average, 26.2 nonaccidental deaths occurred per day, including 12.1 from cardiovascular diseases and 7.2 from respiratory diseases (Table 1). The mean daily average temperature and humidity in Xi’an were 13.4°C and 66.5%, respectively.

**Table 1:** Distribution of daily data on mortality and weather conditions in Xi’an, China (2004–2008).

Percentile				
Mean ± SD	Minimum	25th	50th	75th	Maximum
Daily death counts												
Total nonaccidental		26.2 ± 9.7		4.0		20.0		25.0		31.0		128.0
Cardiovascular		12.1 ± 5.7		0.0		8.0		11.0		15.0		39.0
Respiratory		7.2 ± 3.8		0.0		4.0		7.0		9.0		29.0
Injury		1.8 ± 1.7		0.0		1.0		1.0		3.0		19.0
Weather conditions												
Temperature (°C)		13.4 ± 9.8		–8.0		5.0		14.0		22.0		32.0
Relative humidity (%)		66.5 ± 16.7		15.0		55.0		68.0		79.0		100.0

During 2004–2008, the Xi’an mean daily average concentration of PM_2.5_ was 182.2 µg/m^3^ (Table 2), which was much higher than the WHO Global Guidelines (annual average: 10 µg/m^3^; WHO 2006) and than the reported levels of PM_2.5_ for other Chinese cities such as Beijing (annual average: 122 µg/m^3^; Guo et al. 2009), Shanghai (annual average: 55 µg/m^3^; Kan et al. 2007), and Shenyang (annual average: 75 µg/m^3^; Ma et al. 2011). Meanwhile, the mean daily average concentrations of SO_2_ and NO_2_ were 48.4 and 38.2 µg/m^3^.

**Table 2:** Descriptive statistics for air pollutants in Xi’an, China (2004–2008).

Observation period	Pollutant	Observation (*n*)	Mean ± SD (µg/m^3^)	Minimum	Maximum	IQR (µg/m^3^)	PM_2.5_ mass (%)
1 January 2004–31 December 2008													
		PM_2.5_	1,756		182.2 ± 110.1		16.4		768.6		114.9		—
		SO_2_	1,827		48.4 ± 28.9		8.0		260.0		30.0		—
		NO_2_	1,827		38.2 ± 15.0		6.4		110.0		21.0		—
		OC	1,749		28.3 ± 18.3		5.1		142.3		19.3		15.5
		EC	1,749		12.0 ± 8.3		0.2		84.2		8.8		6.6
		Na^+^	1,649		2.9 ± 1.4		0.0		12.7		1.9		1.6
		NH_4_^+^	1,538		8.8 ± 8.5		0.0		61.1		10.7		4.8
		K^+^	1,616		2.2 ± 2.3		0.0		35.3		1.9		1.2
		Mg^2+^	1,666		0.5 ± 0.3		0.0		3.7		0.3		0.3
		Ca^2+^	730		2.0 ± 2.4		0.0		22.4		1.9		1.1
		F^–^	1,429		0.6 ± 0.3		0.0		3.4		0.5		0.3
		Cl^–^	1,670		5.1 ± 3.5		0.3		32.6		3.6		2.8
		NO_2_^–^	563		0.7 ± 0.4		0.0		3.0		0.4		0.4
		SO_4_^2–^	1,666		31.6 ± 24.4		0.8		198.2		27.8		17.4
		NO_3_^–^	1,644		15.2 ± 12.7		0.0		85.5		15.4		8.4
1 January 2006–31 December 2008													
		S	1,028		5.1 ± 3.5		0.1		24.8		4.3		2.8
		Cl	1,027		1.3 ± 1.6		0.0		11.8		1.5		0.7
		K	1,007		1.8 ± 1.7		0.0		22.5		1.6		1.0
		Ca	904		2.5 ± 3.3		0.0		30.6		2.3		1.4
		Ti	1,026		0.14 ± 0.15		0.00		1.63		0.10		0.08
		Cr	952		0.01 ± 0.01		0.00		0.10		0.01		0.01
		Mn	1,026		0.11 ± 0.08		0.00		0.56		0.09		0.06
		Fe	1,013		1.6 ± 1.7		0.0		20.0		1.3		0.87
		Ni	836		0.01 ± 0.03		0.00		0.55		0.01		0.01
		Zn	1,028		1.4 ± 1.1		0.0		8.6		1.2		0.79
		As	676		0.04 ± 0.03		0.00		0.24		0.03		0.02
		Br	962		0.04 ± 0.05		0.00		0.56		0.04		0.02
		Mo	1,009		0.06 ± 0.05		0.00		0.37		0.03		0.03
		Cd	990		0.03 ± 0.02		0.00		0.13		0.03		0.02
		Pb	1,025		0.50 ± 0.38		0.00		3.13		0.41		0.27

Over the 5 years (1,827 days) of the study, we recorded 1,749 observations of OC and EC; the averaged concentrations were 28.3 µg/m^3^ for OC and 12.0 µg/m^3^ for EC, accounting for 15.5% and 6.6% of the total PM_2.5_ mass, respectively (Table 2). Besides OC and EC, the other largest contributors to PM_2.5_ were SO_4_^2–^ (17.4%), NO_3_^–^ (8.4%), NH_4_^+^ (4.8%), and S (2.8%).

Generally, moderate to high correlations (*r* = 0.5–0.8) were observed for PM_2.5_ with OC, EC, S, Cl, K, Mg^2+^, Cl^–^, K^+^, SO_4_^2–^, NO_3_^–^, and NH_4_^+^ levels [see Supplemental Material, Table 1 (http://dx.doi.org/10.1289/ehp.1103671)]. PM_2.5_ was modestly correlated with Na^+^ levels (*r* = 0.33). Consistent with previous studies (Ostro et al. 2007), Ni levels were weakly correlated with PM_2.5_ (*r* = 0.13) and other constituents.

Figure 1 summarizes the quantitative regression results for single-day lags 0–3 of PM_2.5_ mass and various constituents (before adjusting for PM_2.5_). We found significant associations of PM_2.5_ mass with daily mortality; an IQR increment in the 1-day lagged concentrations of PM_2.5_ (182.2 µg/m^3^) corresponded to a1.8% [95% confidence interval (CI): 0.8%, 2.8%], 3.1% (95% CI: 1.6%, 4.6%), and 4.5% (95% CI: 2.5%, 6.4%) increase of total, cardiovascular, and respiratory mortality, respectively. Consistent with previous studies (Ito et al. 2011; Ostro et al. 2007; Peng et al. 2009), the effect estimates of PM_2.5_ constituents varied by lag structures and mortality outcomes. OC, EC, NH_4_^+^, Cl^–^, NO_3_^–^, Cl, and Ni showed the strongest associations in that more than half of the associations assessed were positive and statistically significant. At least one positive significant association was found for Na^+^, K^+^, Mg^2+^, SO_4_^2–^, S, K, and As. We did not observe positive significant associations for F^–^, Ca, Ti, Cr, Mn, Fe, Zn, Br, Mo, Cd, or Pb [see Supplemental Material, Figure 2 (http://dx.doi.org/10.1289/ehp.1103671)].

**Figure 2:**
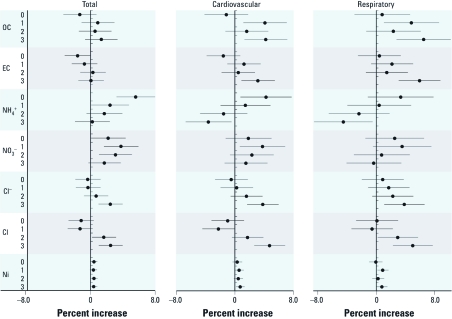
Estimated percent increases [mean (95% CI)] in total, cardiovascular, and respiratory mortality per IQR increase in pollutant concentrations on the current day (lag 0) or the previous 1–3 days (lags 1, 2, and 3), adjusted for PM_2.5_ mass, temporal trend, day of the week, temperature, relative humidity, and SO_2_ and NO_2_ concentrations.

Figure 2 shows the effect estimates of PM_2.5_ constituents (OC, EC, NH_4_^+^, NO_3_^–^, Cl^–^, Cl, and Ni) that were significantly associated with at least one outcome and lag period after further adjustment for PM_2.5_ mass. OC and EC were positively associated with cardiovascular and respiratory mortality (for lags 1–3 and lag 3, respectively), but were not clearly associated with total mortality. NH_4_^+^ and NO_3_^–^ were significantly associated with total and cardiovascular mortality, but not with respiratory mortality. Cl^–^, Cl, and Ni were significantly associated with all three mortality outcomes for at least one lagged exposure. It should be noted that NH_4_^+^ (lag 3) and Cl^–^ (lag 1) were negatively and statistically significantly associated with cardiovascular or respiratory mortality. Na^+^, K^+^, Mg^2+^, SO_4_^2–^, S, K, and As, after adjustment for PM_2.5_, were no longer positively and statistically significantly associated with any of the outcomes, and some of the adjusted associations even became negative and statistically significant [see Supplemental Material, Figure 3 (http://dx.doi.org/10.1289/ehp.1103671)]. Interestingly, after adjusting for PM_2.5_, associations with an IQR increase in NO_3_^–^ were stronger than associations with an IQR increase in PM_2.5_ mass for total and cardiovascular mortality. For instance, for lag 1, an IQR increase in NO_3_^–^ (15.2 µg/m^3^) was associated with 3.8% (95% CI: 1.7%, 5.9%) increase in total mortality, compared with 1.8% (95% CI: 0.8%, 2.8%) for an IQR increase (182.2 µg/m^3^) in PM_2.5_ mass.

**Figure 3:**
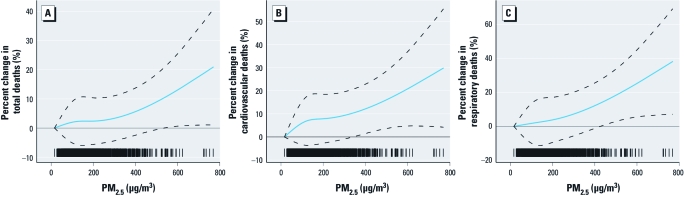
Exposure–response relationships (smoothing plots) of PM_2.5_ against total (*A*), cardiovascular (*B*), and respiratory (*C*) mortality (df**= 3) in Xi’an, adjusted for temporal trend, day of the week, temperature, relative humidity, and SO_2_ and NO_2_ concentrations. The *x*-axis is the PM_2.5_ concentrations (single day lag, L1); the *y*-axis is the estimated percent change in deaths; the solid blue lines indicate the estimated mean percent change in daily death numbers using the lowest PM_2.5_ concentration as the reference level; and the dashed lines represent the 95% CI.

Figure 3 shows the exposure–response relationships for PM_2.5_ mass (single day lag 1) with total, cardiovascular, and respiratory mortality between 2004 and 2008 in Xi’an. For all three mortality outcomes, we observed almost linear relationships, with no evidence of obvious threshold concentrations below which PM_2.5_ had no effect on mortality outcomes. The differences in the deviance between the linear and spline models did not indicate a significant improvement in the fit of the spline versus linear models. In the linear models, a 10-µg/m^3^ increment in the 1-day lagged PM_2.5_ was associated with 0.2% (95% CI: 0.1%, 0.3%), 0.3% (95% CI: 0.1%, 0.4%), and 0.4% (95% CI: 0.2%, 0.6%) increases in total, cardiovascular, and respiratory mortality, respectively.

As expected, deaths due to injury were not associated with PM_2.5_ constituents [there was only 1 significant association out of 92 comparisons when adjusted for PM_2.5_; see Supplemental Material, Table 2 (http://dx.doi.org/10.1289/ehp.1103671)]. Altering the df per year for time trend within a range of 3–10 df did not substantially change the regression results (data not shown).

## Discussion

Evidence obtained in this time–series analysis showed that PM_2.5_ mass and several constituents were associated with total nonaccidental and cardiopulmonary disease-related mortality in Xi’an. The observed levels of PM_2.5_ and its constituents in our study were much higher than earlier health studies of PM_2.5_ constituents in developed countries. Several constituents that were associated with mortality (NH_4_^+^, NO_3_^–^, Cl^–^, OC, EC, Cl) are associated with the combustion of fossil fuels such as coal and heavy oil in Xi’an (Cao et al. 2005, 2009). We found stronger associations for NO_3_^–^ with total and cardiovascular mortality than for PM_2.5_ mass. We did not find evidence of threshold concentrations below which PM_2.5_ was not associated with mortality in Xi’an. To our knowledge, this is the first study of its kind in a developing country to investigate the health effects of PM_2.5_ constituents.

The results of our study in Xi’an indicate considerable risk heterogeneity among the various PM_2.5_ constituents. Consistent with previous epidemiological studies on PM constituents (Ito et al. 2011; Laden et al. 2000; Ostro et al. 2007, 2008; Peng et al. 2009; Zhou et al. 2011), we found that PM_2.5_ constituents resulting from the combustion of fossil fuel (e.g., NH_4_^+^, NO_3_^–^, Cl^–^, OC, EC, Cl, Ni) maintained significant positive associations with mortality outcomes even after we adjusted for PM_2.5_. In contrast, we did not find significant associations between mortality and common crustal elements (e.g., Ca and K) in Xi’an, which is consistent with a previous study performed in six U.S. cities that showed PM_2.5_ crustal particles were not associated with daily mortality (Laden et al. 2000). It should be noted that we observed statistically significant associations for some, but not all, lag structures of PM_2.5_ constituents. Further research is needed to clarify relationships between the timing of exposures and their potential health effects.

Our analysis indicates positive associations of cardiopulmonary mortality with IQR increases in OC or EC during the previous 1–3 days even after adjusting for PM_2.5_ mass. This is consistent with the findings of a meta-analysis of short-term exposure time–series studies of EC and daily mortality that reported positive associations with cardiopulmonary mortality (Smith et al. 2009). The results of a recent cohort study in California suggest that long-term exposure to OC also increase cardiopulmonary mortality (Ostro et al. 2010). Additionally, several previous studies support the biological plausibility of a link between exposure to OC or EC and exacerbations of cardiopulmonary diseases (Gold et al. 2005; Henneberger et al. 2005; Jansen et al. 2005; Lanki et al. 2006; Lewne et al. 2007; Mar et al. 2005; Shih et al. 2008; von Klot et al. 2009). For example, one study in Germany examined weekly electrocardiograms of 56 men with a history of heart disease and found significant associations of OC or EC with changes in myocardial repolarization, which could increase the risk of sudden cardiac death (Henneberger et al. 2005). Gold et al. (2005) found associations of EC with ST-segment depression among a panel of 24 elderly Boston residents. Similarly, Lanki et al. (2006) examined the health effects of five PM_2.5_ components (Si, S, Ni, Cl, and EC), and found only EC had significant association with ST-segment depression in multipolluant models. Exposure to OC or EC was also associated with increased nitric oxide (NO) in exhaled breath, a marker of airway inflammation (Mar et al. 2005). Thus, exposures to both OC and EC are associated with a number of indicators that could contribute to cardiopulmonary mortality.

NO_3_^–^ was positively associated with mortality in our study. To date, only a few epidemiological studies have examined the relationships of NO_3_^–^ with mortality, and their findings were inconclusive. For example, Klemm et al. (2004) found a positive but insignificant association between NO_3_^–^ and mortality in Atlanta (Georgia), whereas Ostro et al. (2007) found a significant association between NO_3_^–^ and mortality in six California counties. More studies are needed to understand the health effects of NO_3_^–^. In our study, SO_4_^2–^ (mean level: 31.6 µg/m^3^) was not associated with mortality, which is consistent with toxicological studies showing little toxic evidence of SO_4_^2–^ effects on the cardiopulmonary system at typical environmental concentrations (Reiss et al. 2007). As Schlesinger and Cassee (2003) pointed out, the minimal effective concentration of SO_4_^2–^ to alter pulmonary mechanical function in normal humans following acute exposure is > 1,000 µg/m^3^.

In our analysis, an IQR increase of 0.01 µg/m^3^ in 1-day lagged Ni was associated with 0.4% (95% CI: 0.0%, 0.8%), 0.6% (95% CI: –0.1%, 1.2%) and 0.9% (95% CI: 0.2%, 1.7%) increases in total, cardiovascular, and respiratory mortality. As a transition metal, Ni may affect health by producing reactive oxygen species and increasing oxidative stress (Lippmann et al. 2006; Schlesinger et al. 2006). In fact, existing epidemiological studies provide evidence of adverse effects for several transition metals (Dominici et al. 2007; Huang et al. 2003; Lippmann et al. 2006; Ostro et al. 2007, 2008). For example, Huang et al. (2003) found that exposure to a factor including vanadium (V), Zn, and copper (Cu) from concentrated ambient particles was associated with increased blood fibrinogen levels. Using the National Morbidity, Mortality, and Air Pollution Study (NMMAPS) database, Lippmann et al. (2006) found that daily mortality rates in the 60 U.S. cities with speciation data were significantly associated with average levels of Ni and V, but not other measured species. In Xi’an, the major source of Ni in PM_2.5_ is fossil fuel combustion, especially heavy oil (Shen et al. 2009b). The role of Ni in PM_2.5_ health hazards should be investigated further.

In our analysis, a 10-µg/m^3^ increment in the 1-day lagged concentrations of PM_2.5_ was associated with 0.2% (95% CI: 0.1%, 0.3%), 0.3% (95% CI: 0.1%, 0.4%), and 0.4% (95% CI: 0.2%, 0.6%) increases in total, cardiovascular, and respiratory mortality, respectively. Compared with studies of PM_2.5_ and daily mortality in developed countries (Franklin et al. 2007; Ostro et al. 2006; Ueda et al. 2009; Zanobetti and Schwartz 2009), our estimations of the associations of PM_2.5_ with mortality were somewhat lower in magnitude per amount of PM_2.5_ mass. For example, a multicity analysis in 112 U.S. cities found that a 10-µg/m^3^ increase in PM_2.5_ was associated with a 1.0% increase in total mortality, a 0.9% increase in cardiovascular mortality, and a 1.7% increase in respiratory mortality (Zanobetti and Schwartz 2009), whereas our findings are in agreement with earlier evidence (Aunan and Pan 2004) suggesting weaker associations between health outcomes and unit increases in air pollution exposures in China than in developed countries. This may be explained by differences in the composition and toxicity of PM, as well as differences in local PM concentrations and population sensitivity to PM in addition to differences in age structure and other population characteristics. Lower risks of death per unit increases in pollutants when concentrations are high may reflect the selective attrition of vulnerable members of the population who die before concentrations reach the maximum level (Wong et al. 2008). Also, associations between mortality and PM exposures ranging from low (e.g., exposure levels associated with ambient air pollution) to high (e.g., exposure levels associated with cigarette smoking) concentrations suggest that the exposure–response curve of PM often tends to become flat at higher concentrations (Pope et al. 2009).

Accurate information on the shape of exposure–response relationships is crucial for public health assessment, and the demand for providing the relevant curves has been growing (Dominici et al. 2002). Dose–response relationships may vary by location depending on factors such as the air pollution mixture, climate, and overall health of the studied population (Samoli et al. 2005). In our study population, we did not observe evidence for a threshold concentration below which PM_2.5_ was not associated with mortality, suggesting that linear models without a threshold are appropriate for assessing the effect of PM_2.5_ on daily mortality for the high-exposure settings typical of developing countries.

Our study has limitations. First, we evaluated the associations of multiple constituents and lags with three different mortality outcomes; some significant associations, therefore, may have occurred by chance. Second, because of moderate-to-high collinearity among PM_2.5_ constituents, we could not adjust for multiple exposures, and some associations may reflect the effects of other correlated components. We did not measure several elements such as selenium (Se), V, and silicon (Si), although previous studies reported significant associations between these elements and adverse health outcomes (Laden et al. 2000; Ostro et al. 2007), and we could not evaluate ozone (O_3_) due to a lack of monitoring data in Xi’an. As in many previous time–series studies, we used PM_2.5_ monitoring results from a fixed station as a proxy measure for population exposures to air pollution. As a result, a number of issues may arise given that ambient monitoring results differ from personal exposure level to air pollutants (Sarnat et al. 2001, 2005). In addition, variation in the extent of exposure misclassification among individual constituents may influence associations. Finally, we did not conduct formal source apportionment of PM_2.5_ constituents, and therefore cannot identify the source components that contributed most to the associations between PM_2.5_ and mortality.

## Conclusions

Our findings suggest that PM_2.5_ constituents from fossil fuel combustion may have an appreciable influence on the health effects attributable to PM_2.5_. Associations of PM_2.5_ with mortality in Xi’an are somewhat lower in magnitude per amount of PM_2.5_ mass compared with associations reported for populations in developed countries. Our findings add support to previously reported evidence of PM_2.5_-related health effects in China and suggest that combustion-associated pollutants are particularly important.

## Supplemental Material

(53 KB) PDF
