# Supplementary material for: Fine Particulate Matter Constituents and Cardiopulmonary Mortality in a Heavily Polluted Chinese City
Source: Environ Health Perspect. 2012 Jan 3;120(3):373–8. doi: 10.1289/ehp.1103671 (PMC3295342; doi:10.1289/ehp.1103671)
Supplement: (53 KB) PDF [file ehp.1103671.s001.pdf]

Supplemental Material

Fine Particulate Matter Constituents and Cardiopulmonary Mortality in a Heavily Polluted Chinese City

Junji Cao, Hongmei Xu, Qun Xu, Bingheng Chen, Haidong Kan

Supplemental Material, Figure 1: Page 3

Supplemental Material, Figure 2: Page 4

Supplemental Material, Figure 3: Page 5

Supplemental Material, Table 1: Pages 6

Supplemental Material, Table 2: Page 7

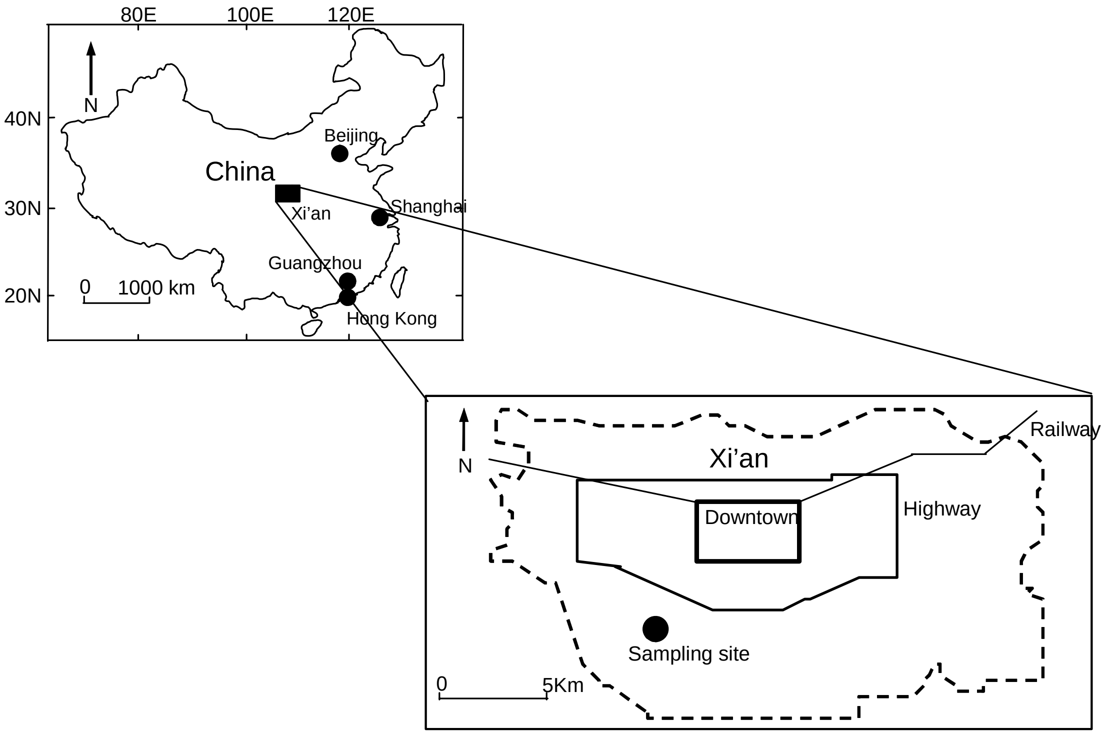

Figure 1. Location of sampling site of $PM_{2.5}$ in Xi'an. The study area is within the dotted line.

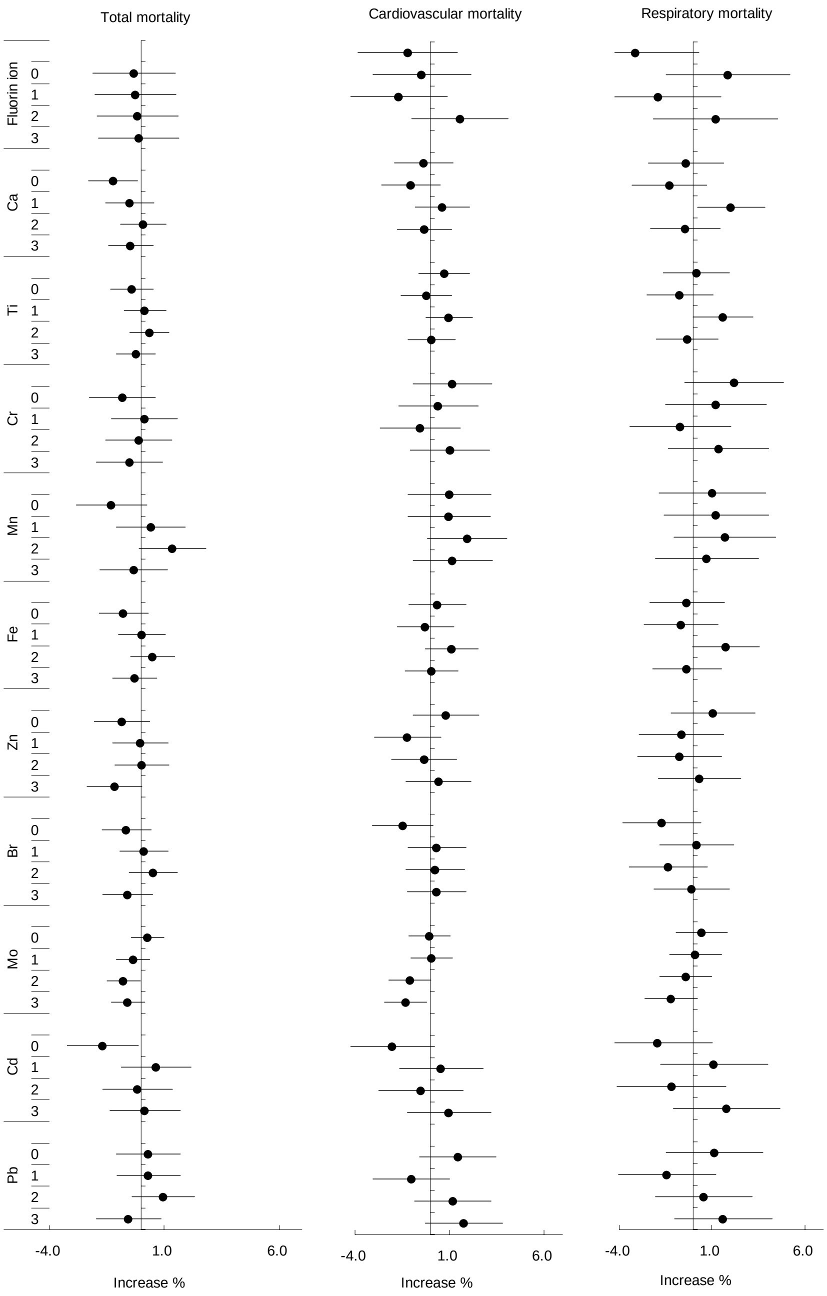

Figure 2. Estimated percent increase [mean (95% CI)] in mortality per IQR increase in pollutant concentrations on the current day (lag 0) or the previous 1-3 days (lags 1, 2 and 3), adjusted for temporal trend, day of the week, temperature, relative humidity, and SO_2 and NO_2 concentrations

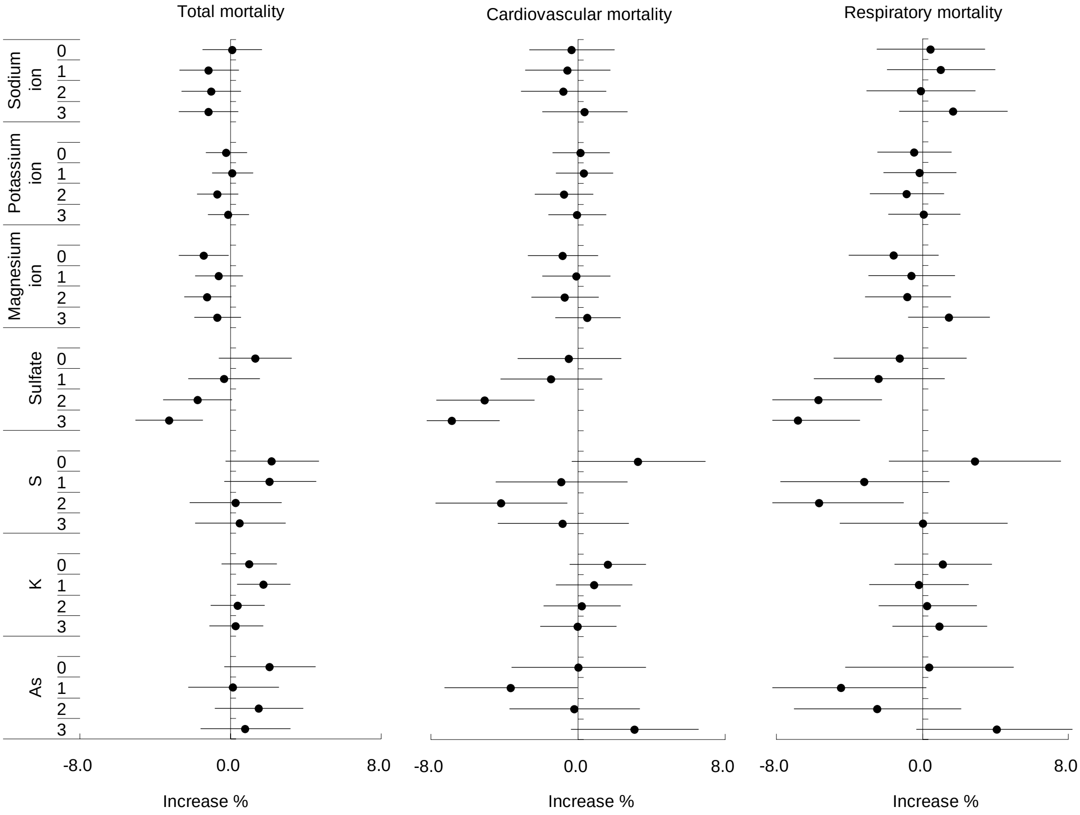

Figure 3. Estimated percent increase [mean (95% CI)] in mortality per IQR increase in pollutant concentrations on the current day (lag 0) or the previous 1-3 days (lags 1, 2 and 3), adjusted for $PM_{2.5}$ mass, temporal trend, day of the week, temperature, relative humidity, and SO_2 and NO_2 concentrations

Table 1. Correlations among $PM_{2.5}$ mass and selected constituents

	$PM_{2.5}$	OC	EC	SO_4^{2-}	NO_3^-	S	Cl	K	Ni	Na^+	NH_4^+	K^+	Mg^{2+}	Cl^-
$PM_{2.5}$	1													
OC	0.70	1												
EC	0.66	0.87	1											
SO_4^{2-}	0.76	0.56	0.52	1										
NO_3^-	0.81	0.68	0.63	0.85	1									
S	0.66	0.67	0.64	0.81	0.76	1								
Cl	0.54	0.78	0.74	0.22	0.40	0.45	1							
K	0.54	0.61	0.49	0.31	0.39	0.52	0.63	1						
Ni	0.13	0.05	0.04	0.14	0.10	0.02	0.05	0.05	1					
Na^+	0.33	0.20	0.10	0.28	0.27	0.22	0.32	0.29	0.28	1				
NH_4^+	0.79	0.61	0.52	0.93	0.89	0.84	0.32	0.33	0.08	0.25	1			
K^+	0.67	0.61	0.48	0.63	0.62	0.65	0.49	0.80	0.20	0.19	0.63	1		
Mg^{2+}	0.52	0.45	0.43	0.26	0.31	0.27	0.46	0.70	0.25	0.40	0.17	0.46	1	
Cl^-	0.75	0.83	0.74	0.47	0.62	0.49	0.88	0.63	0.11	0.34	0.53	0.62	0.52	1

Table 2. Summary of statistically significant associations between injury mortality and $PM_{2.5}$ constituents with alternative pollutant lags (L) (numbers in the table indicate whether single lags of 0–3 days were statistically significant)

Pollutants	Before adjustment for $PM_{2.5}$ mass	After adjustment for $PM_{2.5}$ mass
K^+	L1*	-
SO_4^{2-}	L2*	-
Cl^-	-	L1*
$PM_{2.5}$ mass, OC, EC, Na^+, NH_4^+, Mg^{2+}, NO_3^-, Ca^{2+}, F^-, NO_2^-, Ca, Ti, Cr, Mn, Fe, Zn, Br, Mo, Cd, Pb	-	-

* $p < 0.05$
